# Case Report: Immune reconstitution–related neurological deterioration in advanced HIV infection with multiple opportunistic infections: a diagnostic challenge

**DOI:** 10.3389/fmed.2026.1742917

**Published:** 2026-02-23

**Authors:** Jesús Endara-Mina, Cristopher-Josué Escudero, Victor Samaniego, Karla Fuentes, William Tapia, Cesar Intriago

**Affiliations:** 1 Facultad de Ciencias Jurídicas y Políticas, Universidad Técnica Particular de Loja (UTPL), Loja, Ecuador; 2 Coordinación de Docencia e Investigación, Hospital Provincial General Pablo Arturo Suárez, Ángel Ludeña s/n, Quito, Ecuador; 3 Facultad de Ciencias Médicas, Universidad Central del Ecuador (UCE), Quito, Ecuador; 4 Servicio de Medicina Interna, Hospital de Especialidades Eugenio Espejo, Quito, Ecuador; 5 Facultad de Ciencias de la Salud Eugenio Espejo, Universidad Técnica Equinoccial (UTE), Quito, Ecuador

**Keywords:** AIDS, antiretroviral agents, HIV, syphilis, toxoplasmosis, *Mycobacterium tuberculosis*

## Abstract

Immune reconstitution inflammatory syndrome (IRIS) is a serious complication following antiretroviral therapy (ART) initiation in patients with advanced HIV infection, particularly when the central nervous system is involved and multiple opportunistic infections coexist. We report the case of a 26-year-old man with newly diagnosed advanced HIV infection who developed rapid neurological deterioration shortly after ART initiation during hospitalization. Neuroimaging revealed a necrotic central nervous system mass lesion with extensive edema and mass effect. Serological testing demonstrated prior exposure to *Toxoplasma gondii* and active *Treponema pallidum* infection. Despite broad antimicrobial therapy, corticosteroids, and supportive care, the patient experienced progressive clinical deterioration and died. Retrospective reassessment of the clinical course, imaging findings, epidemiological context, and treatment response suggested an IRIS-related inflammatory process, with central nervous system tuberculosis–associated IRIS representing the most plausible underlying mechanism, while toxoplasmosis and syphilis were considered potential concomitant or confounding conditions. This case underscores the diagnostic complexity of IRIS in advanced HIV infection and highlights the importance of a cautious, probabilistic, and evidence-based approach to avoid etiologic misclassification in severe neurological presentations.

## 1 Introduction

Immune reconstitution inflammatory syndrome (IRIS) is a recognized complication of antiretroviral therapy (ART) in people living with HIV and may significantly impact morbidity and quality of life, particularly in patients with advanced immunosuppression (1). Although ART aims to restore immune function and reduce HIV-related complications, immune recovery may paradoxically trigger an exaggerated inflammatory response against opportunistic pathogens, leading to clinical deterioration rather than improvement.

IRIS remains a partially understood entity and typically develops within the first months after ART initiation in patients with Human Immunodeficiency Virus/Acquired Immunodeficiency Syndrome (HIV/AIDS) (2). Clinically, it may present as paradoxical worsening of a previously diagnosed and treated opportunistic infection or as unmasking of a previously subclinical condition. These two patterns paradoxical and unmasking IRIS are useful conceptual frameworks; however, in real-world clinical settings, especially in patients with profound immunosuppression or incomplete baseline investigations, strict classification may be challenging and of limited practical value (2).

A broad range of conditions have been associated with IRIS, most commonly *Mycobacterium tuberculosis*, *Cryptococcus neoformans*, Kaposi’s sarcoma, non-Hodgkin lymphoma, and progressive multifocal leukoencephalopathy (3). Among these, tuberculosis represents the most frequent and clinically significant trigger of IRIS worldwide, particularly in regions where late HIV diagnosis is common. Central nervous system involvement is among the most severe manifestations and is associated with high morbidity and mortality.

Cerebral toxoplasmosis, caused by *Toxoplasma gondii*, is a common opportunistic infection in advanced HIV infection but represents an uncommon cause of IRIS. This rarity has been attributed to the parasite’s immune evasion mechanisms and the effectiveness of modern ART. Consequently, toxoplasmosis-associated IRIS has been described primarily in isolated case reports and small series, and its clinical spectrum remains poorly defined (3, 4).

Similarly, coinfection with *Treponema pallidum* in people living with HIV may follow an atypical and accelerated course. Reported manifestations include sudden neurological deterioration, early progression to neurosyphilis, atypical or seronegative serologic responses, and suboptimal response to standard benzathine penicillin therapy (5, 6). Although syphilis-associated IRIS has been reported, it remains infrequent and is most often characterized by cutaneous or ocular involvement rather than fulminant central nervous system disease.

Taken together, these considerations highlight the diagnostic complexity of IRIS in patients with advanced HIV infection and multiple overlapping opportunistic infections. Careful integration of clinical evolution, epidemiological context, radiological findings, and therapeutic response is essential to avoid etiologic misclassification and to guide appropriate clinical management in severe neurological presentations.

## 2 Aim of the study

The aim of this case report is to describe a severe neurological deterioration occurring after antiretroviral therapy initiation in a patient with advanced HIV infection and multiple opportunistic infections, and to highlight the diagnostic challenges associated with IRIS in this context. Specifically, this report seeks to emphasize the difficulty of etiologic attribution when central nervous system involvement, overlapping infections such as toxoplasmosis and syphilis, and limited baseline immunologic data coexist, underscoring the need for a cautious, probabilistic, and clinically grounded approach to IRIS evaluation.

## 3 Methods

### 3.1 Study design and reporting standards

This manuscript describes a single-patient clinical case and was prepared in accordance with the CARE (CAse REport) guidelines to ensure transparent, complete, and structured reporting (7). The CARE checklist was used during manuscript preparation to guide inclusion of essential clinical information.

### 3.2 Data sources and collection

Clinical data were retrospectively collected from the patient’s medical records, including demographic characteristics, presenting symptoms, physical examination findings, laboratory and microbiological results, imaging studies, therapeutic interventions, and clinical outcomes. Due to the retrospective nature of the case and rapid clinical deterioration, some elements of the clinical timeline could not be fully reconstructed; these limitations are explicitly acknowledged.

### 3.3 Chronology and clinical assessment

Particular attention was given to the temporal relationship between ART initiation, antimicrobial treatments, corticosteroid administration, and neurological deterioration, as chronology is central to the evaluation of IRIS. When exact dates were unavailable, clinical events were described using relative hospital days to preserve chronological clarity without introducing unsupported assumptions.

### 3.4 Diagnostic framework

IRIS was approached as a clinical syndrome characterized by inflammatory deterioration temporally associated with immune recovery after ART initiation. Definitions of paradoxical and unmasking IRIS were derived from previously published criteria; however, strict classification into either category was not enforced due to incomplete baseline immunologic data and the presence of multiple overlapping opportunistic infections (8). Diagnostic reasoning relied on integrated clinical, radiological, laboratory, and epidemiological assessment, and etiologic attribution was expressed using probabilistic terminology rather than definitive diagnoses.

### 3.5 Laboratory and imaging investigations

Laboratory evaluation included routine hematological and biochemical tests, HIV viral load quantification, and CD4 + T-cell enumeration when available. Serologic testing for *Toxoplasma gondii* and *T. pallidum* was performed according to institutional protocols. Tuberculosis evaluation consisted of screening tests routinely used in clinical practice, interpreted cautiously in the context of advanced HIV infection.

Chest computed tomography (CT) and brain CT and magnetic resonance imaging (MRI) were reviewed descriptively and interpreted in clinical context. Imaging findings were not considered diagnostic in isolation but were integrated with clinical evolution and laboratory data.

### 3.6 Therapeutic interventions

Therapeutic management, including antimicrobial therapy, antituberculous treatment, corticosteroids, anticonvulsants, and ART, was documented as recorded in the medical records. Changes in therapy were described when available. Where the precise timing or rationale of interventions could not be fully determined, this was explicitly stated to avoid overinterpretation.

### 3.7 Ethical considerations

This case report was conducted in accordance with national ethical regulations. In line with local legislation, single case reports do not require approval from a research ethics committee when patient confidentiality is preserved. Written informed consent for publication was obtained from the patient’s legal representative, and all identifying information was removed prior to analysis.

## 4 Case presentation

### 4.1 Patient information

A 26-year-old man with a recent diagnosis of advanced HIV infection was admitted to a tertiary care hospital in February 2025 due to progressive neurological symptoms accompanied by persistent respiratory complaints. No chronic medical conditions were documented prior to the HIV diagnosis. There was no known family history of neurological, genetic, or autoimmune diseases.

Information regarding psychosocial background was limited due to the acute severity of the clinical presentation. The available medical records did not document substance use, occupational exposures, or other relevant social determinants of health. No opportunistic infections had been formally diagnosed before the current admission.

### 4.2 Clinical presentation

The patient reported a two-week history of fever, nocturnal productive cough, diaphoresis, and diffuse headache. On physical examination at admission, he appeared ill with mild respiratory distress. Pulmonary auscultation revealed bilaterally reduced breath sounds without focal crackles or wheezing.

Neurological examination at presentation showed the patient to be alert and oriented, with no focal neurological deficits. Cranial nerve examination was unremarkable, and motor and sensory functions were preserved.

### 4.3 Initial investigations

Initial laboratory evaluation demonstrated lymphopenia. Renal and hepatic function tests were within normal limits. HIV-1 viral load was markedly elevated (2,630,000 copies/mL). Serologic testing for hepatitis B and C was negative. Tuberculosis screening tests were negatives. Sputum samples were analyzed for tuberculosis using GeneXpert and smear microscopy, with negative results in both tests. Histoplasmosis score was realizaed resulting negative and confirmed with Lipoarabinomannan in urine negative.

**Figure 1:**
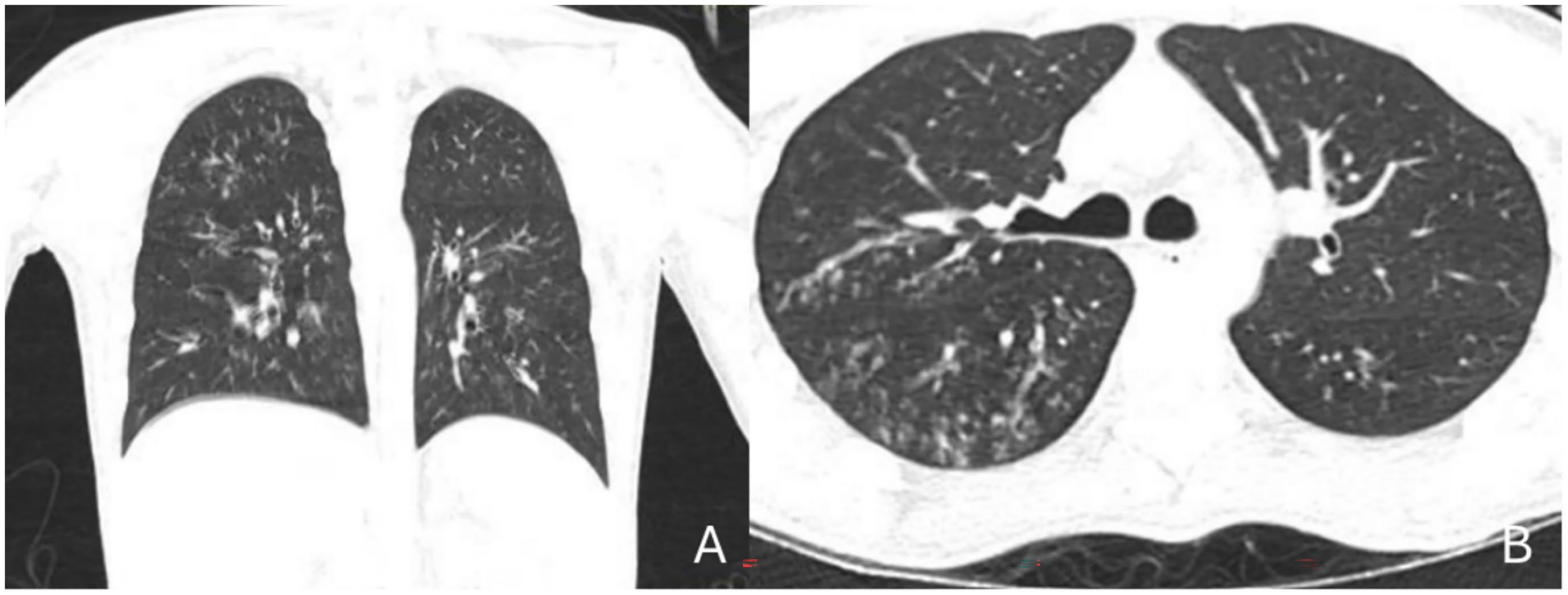
Chest computed tomography (CT). **(A)** Coronal view; **(B)** Axial view: **(A)** Symmetrical bilateral involvement is confirmed, showing a ground-glass opacity pattern with interstitial thickening, predominantly affecting the middle and lower lobes. No pleural effusions or visible mediastinal masses are observed. This coronal view highlights the classic “inverted butterfly wings” distribution pattern, characteristic of immunosuppressed patients. **(B)** Bilateral ground-glass opacities are noted, suggestive of atypical interstitial pneumonia, highly compatible with infective process in an immunocompromised patient. The pattern is centrally and peribronchially predominant, accompanied by bronchial wall thickening and a fine reticulonodular appearance. No lobar consolidations, cavitations, or mediastinal lymphadenopathy are identified.

Microbiological studies of respiratory samples identified carbapenemase-producing *Pseudomonas aeruginosa*. Chest CT revealed bilateral diffuse ground-glass opacities with interstitial involvement, predominantly affecting the middle and lower lung fields, without pleural effusion or mediastinal lymphadenopathy (Figure 1).

### 4.4 Initial management

Based on clinical, radiological, and microbiological findings, empirical antimicrobial therapy with trimethoprim–sulfamethoxazole and colistin was initiated. ART was initiated early during hospitalization, in accordance with standard national recommendations for advanced HIV infection. Exact timing relative to admission could not be fully reconstructed from the available records.

### 4.5 Neurological deterioration

**Figure 2:**
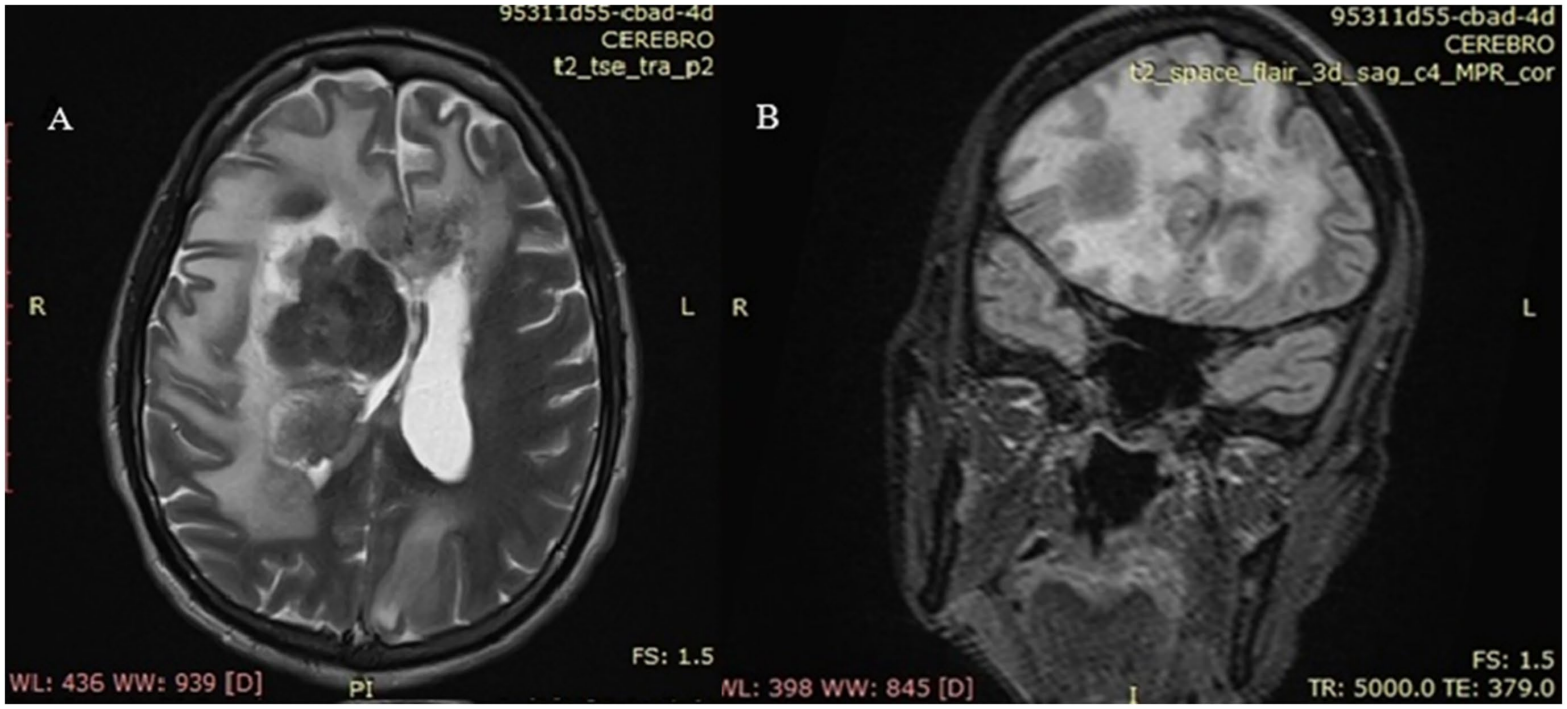
Brain magnetic resonance imaging. **(A)** Axial view – T2-weighted sequence; **(B)** Coronal view – FLAIR sequence: **(A)** A heterogeneous lesion is observed in the right cerebral hemisphere, centered in the lenticulocapsular region, with peripheral T2 hyperintense areas (edema) and a hypointense center, consistent with necrosis. There is collapse of the right frontal horn, displacement of the septum pellucidum, and compression of the ventricular system. No overt transtentorial or subfalcine herniation is evident in this section, although there is marked mass effect. **(B)** A centrally hypointense space-occupying lesion with necrosis, edema, and mass effect is noted. A hyperintense halo is seen in the right basal ganglia region, extending toward the internal capsule and adjacent thalamus, with irregular margins and surrounded by extensive vasogenic edema involving the right cerebral hemisphere. Compression of the frontal horn of the right lateral ventricle and mild leftward midline shift are identified. The perilesional hyperintense pattern on FLAIR suggests a lesion with central necrosis and fluid content, surrounded by edema. The axial T2-weighted image complements the FLAIR view, clearly demonstrating the cystic/necrotic structure with surrounding edema.

Approximately 48 h after admission, the patient developed acute neurological symptoms, including diplopia, nausea, and photophobia, followed by seizures and progressive impairment of consciousness. A brain CT scan demonstrated a large hypodense lesion in the right frontal region, associated with significant mass effect, compression of the right lateral ventricle, and midline shift (Figure 2). Lumbar puncture revealed clear cerebrospinal fluid with elevated protein concentration; no microorganisms were identified using routine diagnostic methods, including genetic and staining tests for Tuberculosis, and serologic panel for viral infections.

Antituberculous therapy was started due to clinical context and epidemiological risk, despite the absence of microbiological confirmation.

### 4.6 Further investigations and disease progression

High-dose corticosteroid therapy (dexamethasone 8 mg every 12 h) and anticonvulsant treatment were initiated to manage cerebral edema and seizures. Follow-up brain magnetic resonance imaging (MRI) showed a heterogeneous necrotic space-occupying lesion centered in the right lenticulocapsular region, surrounded by extensive vasogenic edema and marked ventricular compression.

Repeat laboratory testing revealed a CD4 + T-cell count of 38 cells/μL and a persistently elevated HIV viral load (844,000 copies/mL). Serologic testing was positive for *T. gondii* IgG and IgM antibodies, indicating prior exposure, although these results were interpreted cautiously due to limited specificity for active disease in advanced HIV infection. Serologic testing for *T. pallidum* was reactive, with a VDRL titer of 1:16 and a quantitative value of 331 IU, consistent with active syphilis infection.

Antimicrobial therapy was intensified with high-dose trimethoprim–sulfamethoxazole administered every 8 h, along with antifungal therapy and escalation of corticosteroid treatment to methylprednisolone 125 mg every 12 h. Adjustments in therapy were made in response to ongoing clinical deterioration; however, the precise timing and rationale for some changes could not be fully determined from the available records.

### 4.7 Outcome and follow-up

Despite aggressive antimicrobial therapy, corticosteroids, antiretroviral treatment, and supportive care, the patient continued to deteriorate neurologically and respiratory. He was transferred to a higher-level care facility, where his clinical condition worsened rapidly. After multidisciplinary discussion and in light of the poor prognosis, a palliative approach was adopted in accordance with the family’s wishes. The patient subsequently died.

### 4.8 Patient perspective

The patient perspective could not be obtained due to rapid clinical deterioration and subsequent death.

## 5 Discussion

IRIS remains one of the most challenging complications following ART initiation in patients with advanced HIV infection. CNS involvement represents one of the most severe and life-threatening manifestations, particularly when multiple opportunistic infections coexist and baseline immunologic data are incomplete. This case illustrates the diagnostic complexity of IRIS attribution in real-world clinical settings and the risk of etiologic misclassification when overlapping infections are present.

The diagnosis of IRIS relies on the temporal association between ART initiation and clinical deterioration, in the absence of alternative explanations such as drug toxicity, treatment failure, or natural disease progression (9). In the present case, strict fulfillment of all major and minor diagnostic criteria was not possible due to the absence of baseline CD4 + lymphocyte counts. Nevertheless, the close temporal relationship between ART initiation and rapid neurological worsening, together with the exclusion of more plausible alternative causes, supports the presence of an IRIS-related inflammatory process.

Tuberculosis is the most frequent cause of IRIS worldwide and remains the leading etiology of severe IRIS-related morbidity and mortality, particularly in low- and middle-income countries where late HIV diagnosis is common (10). CNS tuberculosis–associated IRIS is well recognized and may present with intracranial mass lesions, extensive vasogenic edema, raised intracranial pressure, and rapid neurological decline. Importantly, negative initial tuberculosis screening tests do not exclude active or disseminated disease in patients with advanced HIV infection, as diagnostic sensitivity is markedly reduced in this population (10, 11).

In the present case, several features favor central nervous system tuberculosis–associated IRIS as the most plausible underlying mechanism. These include the initial systemic and respiratory presentation, pulmonary imaging findings, the epidemiological context, the temporal relationship between initiation of antiretroviral therapy and subsequent neurological deterioration, as well as the lack of sustained clinical improvement despite high-dose corticosteroid therapy. However, the absence of confirmatory results from the diagnostic tests performed precludes definitive attribution of a single etiological agent. Taken together, these elements are more consistent with previously described patterns of CNS TB–IRIS; nevertheless, the increasing number of reported IRIS cases associated with other microorganisms supports the possibility of coinfection with additional related etiological agents (4, 10).

Cerebral toxoplasmosis remains a common cause of focal brain lesions in advanced HIV infection; however, toxoplasmosis-associated IRIS is rare and remains poorly characterized (12, 13). Notwithstanding, the growing number of reported cases suggests that this etiology may represent an emerging cause of IRIS in this population. In the present case, the diagnosis of cerebral toxoplasmosis met criteria for a possible diagnosis, based on compatible neuroimaging findings and positive serum IgG serology (13). IgG positivity reflects prior exposure rather than active disease, and IgM testing lacks specificity in advanced HIV infection due to immune dysregulation (14). Furthermore, the neuroimaging characteristics of toxoplasmosis substantially overlap with those of CNS tuberculosis and other inflammatory processes, thereby limiting diagnostic specificity (4, 14). Consequently, toxoplasmosis was considered a potentially coexisting condition which, although it could not be established as the primary cause, may be interpreted as a latent coinfection unmasked.

Syphilis represents an additional diagnostic confounder in patients with HIV infection. Coinfection with *T. pallidum* may lead to atypical clinical courses, including early neurological involvement and serologic variability (15, 16). Syphilis-associated IRIS has been described, most commonly presenting with cutaneous or ocular manifestations, particularly in secondary syphilis (17, 18). Although immune reconstitution may exacerbate inflammatory responses to *T. pallidum*, fulminant CNS mass lesions remain rare. In this case, active syphilis may have contributed to immune activation but was unlikely to fully account for the radiological findings or the rapid fatal course.

An important consideration highlighted by this case is the challenge of classifying IRIS as paradoxical or unmasking. While these categories are conceptually useful, strict classification may be inappropriate in patients with advanced immunosuppression, incomplete baseline investigations, and multiple concurrent infections (8, 9). Current International AIDS Society (IAS) and expert consensus recommendations emphasize a pragmatic, clinically driven approach to IRIS diagnosis, prioritizing temporal relationships, disease evolution, and treatment response over rigid categorical definitions (10, 11, 19).

From a clinical perspective, this case underscores the potential consequences of misattributing neurological deterioration to rare IRIS etiologies, such as toxoplasmosis-associated IRIS, while underrecognizing more frequent and severe causes such as CNS tuberculosis–associated IRIS. Early recognition, careful diagnostic reassessment, and close neurological monitoring are essential to improve outcomes in patients with advanced HIV infection initiating ART.

### 5.1 Strengths and limitations

The main strength of this case report lies in the detailed clinical and radiological documentation of a rapidly progressive CNS presentation following ART initiation in advanced HIV infection. The case reflects real-world diagnostic challenges and highlights important pitfalls in IRIS attribution, particularly in the presence of multiple opportunistic infections.

However, several limitations must be acknowledged. The retrospective nature of the report and the absence of baseline CD4 + lymphocyte counts limited the ability to fully characterize immunologic recovery. Definitive microbiological or histopathological confirmation of all potential etiologies was not feasible due to rapid clinical deterioration. The exact timing and rationale of some therapeutic interventions could not be fully reconstructed from the available records. Additionally, a patient perspective could not be obtained. These limitations preclude definitive etiologic conclusions and necessitate a cautious, probabilistic interpretation grounded in clinical plausibility and existing evidence.

## 6 Conclusion

Neurological deterioration following antiretroviral therapy initiation in patients with advanced HIV infection represents a complex and high-risk clinical scenario, particularly when multiple opportunistic infections coexist. This case highlights how immune reconstitution–related inflammatory processes may mimic, overlap with, or exacerbate underlying central nervous system infections, making etiologic attribution challenging in routine clinical practice.

Although cerebral toxoplasmosis and syphilis were considered as possible concomitant conditions, the overall clinical course, imaging findings, epidemiological context, and therapeutic response were most consistent with a central nervous system tuberculosis–associated IRIS. Importantly, negative initial screening tests and the presence of alternative opportunistic infections should not preclude consideration of tuberculosis as a leading cause of IRIS in severely immunosuppressed patients.

This report underscores the importance of careful timing of antiretroviral therapy, close neurological monitoring, and a cautious, probabilistic, and evidence-based diagnostic approach when managing advanced HIV infection. Early recognition of IRIS and awareness of diagnostic pitfalls are essential to reduce misclassification, guide appropriate management, and potentially improve outcomes in severe central nervous system presentations.

## Data Availability

The data are available upon request for ethical reasons. The patient’s medical record is kept in the archives of the Internal Medicine Service at Eugenio Espejo Hospital, Quito, Ecuador. Requests to access these datasets should be directed to jvendara@utpl.edu.ec.
